# Posterior urethral valves: Should we have objective criteria on the adequacy of fulguration?

**DOI:** 10.4103/0971-9261.71742

**Published:** 2010

**Authors:** K. L. N. Rao

**Affiliations:** Editor-in-Chief, JIAPS, Professor and Head, Department of Pediatric Surgery, Advanced Pediatric Center, Post Graduate Institute of Medical Education and Research, Chandigarh 160012, India E-mail: klnrao@hotmail.com

Posterior urethral valves (PUV) are one of the challenging anomalies in children for us to treat. The severity of the disease may vary enormously from a simple obstruction, easy to ablate with a normal life span to renal dysplasia and end stage renal failure requiring renal transplantation. Various mediator issues like vesicoureteral reflux, urinary tract infections, nonfunctioning kidneys, small bladders, voiding dysfunctions, hypertension, stunted growth, etc., require a multidisciplinary approach and dedicated life long care of these children.

Primary renal dysplasia cannot be altered by the surgeon. However progressive renal deterioration after birth should be avoided by the treating clinician, with appropriate management.

A micturating cystourethrogram (MCUG) is the gold standard investigation for the diagnosis of PUV. Endoscopic primary valve ablation is the best initial surgical treatment of valves. This should result in improved stream and reversal of posterior urethral dilatation to normal or near normalcy. After valve fulguration, though it is recommended to repeat an MCUG to check the adequacy of valve fulguration, there are hardly any series reported in the literature where this has been performed routinely. Most of the surgeons are content with an enquiry regarding the stream of urine and presume adequate fulguration and attribute continuing problems of persistent or deteriorating hydroureteronephrosis to the natural course of the disease like valve bladder syndrome or vesicoureteral junction obstruction. Not many surgeons would try to check whether their own fulguration is adequate or not! Personal experience of inadequately treated PUVs, while caring for a high number of children referred to our tertiary health care facility for 60 million people, supports this hypothesis.

It is usually presumed that dilatation of posterior urethra persists for a long period of time even after successful valve fulguration, without much substantiating evidence. It is common sense that where dilatation of any viscera has resulted from a distal obstruction to the flow of contents, adequate relief of obstruction should lead to near normalcy in a great majority. Scientific approach also demands such checks as part of the management.

A simple repeat MCUG a few months post-fulguration, would go a long way in clarifying the issue. Can we assess and document the adequacy of fulguration by comparing the pre and post-fulguration MCUG? Can we avoid the voiding problems and most of the valve bladder syndromes by proven adequate fulguration rather than presumption of adequacy? Can we think of guidelines for a second fulguration? A study conducted at Post Graduate Institute of Medical Education and Research, Chandigarh, India, and published in this issue of the Journal by Menon *et al*. addresses these problems to some extent.

It is expected that this study would provoke thoughts in the minds of all surgeons who are caring for the children suffering from PUVs.

